# Coffee Consumption among Adults in the United States by Demographic Variables and Purchase Location: Analyses of NHANES 2011–2016 Data

**DOI:** 10.3390/nu12082463

**Published:** 2020-08-16

**Authors:** Colin D. Rehm, Joseph C. Ratliff, Claudia S. Riedt, Adam Drewnowski

**Affiliations:** 1 Department of Epidemiology & Population Health, Albert Einstein College of Medicine, Montefiore Medical Center, Bronx, NY 10595, USA; colin.rehm@gmail.com; 2 Keurig Dr Pepper, 5301 Legacy Drive, Plano, TX 75024, USA; joseph.ratliff@kdrp.com (J.C.R.); Claudia.Riedt@kdrp.com (C.S.R.); 3 Center for Public Health Nutrition, University of Washington, Box 353410, Seattle, WA 98195, USA

**Keywords:** coffee, demographics, socioeconomic status, diet quality, caffeine, Nutrient-Rich Foods (NRF9.3), Healthy Eating Index 2015, purchase location

## Abstract

Coffee, obtained from various sources, is consumed by most United States adults. The present analyses of one and two 24-h dietary recalls for 14,865 persons aged ≥20 years in the 2011–2016 National Health and Nutrition Examination Survey (NHANES 2011–2016) aimed to identify socio-demographic predictors of coffee consumption and to examine whether coffee purchase locations differed by population sub-group. Given the emphasis on food and beverage consumption patterns, the relation between coffee consumption and compliance with the Dietary Guidelines of Americans was also examined. Coffee was consumed by 59% of the sample (n = 8551). Survey-adjusted mean intake among consumers was 544.7 g/day. Percent consumers and mean amounts consumed were highest among adults aged 51–70 years (*p* < 0.001), higher income groups (*p* < 0.001), and non-Hispanic Whites (*p* < 0.001). About 74% of coffee consumers obtained their coffee from stores, 9.8% from fast food restaurants, 4.3% from convenience stores, and 4.2% from someone else. Coffee source locations also varied by age, education, income, and race/ethnicity. Coffee consumers had significantly higher Healthy Eating Index (HEI-2015) and higher Nutrient-Rich Foods (NRF9.3) scores in energy-adjusted models and significantly higher HEI 2015 scores in multivariable models. In multivariable models, coffee consumers had diets with less added sugar (*p* < 0.001) but slightly more fat (of all types, including monounsaturated (MUFA), polyunsaturated (PUFA), saturated and solid fats), cholesterol, and alcohol. Their diets had more potassium and magnesium (*p* < 0.001) but less vitamin C (*p* < 0.001). Mean caffeine consumption was 233 mg/day for consumers and 72.3 mg/day for non-consumers. Coffee consumption patterns in the US vary across socio-demographic groups.

## 1. Introduction

Coffee and tea, along with plain drinking water, are among the most frequently consumed beverages in the world [1]. Analyses of the National Health and Nutrition Examination Survey (NHANES) data for years 2003–2012 showed that 75% of US adults aged ≥20 years consumed coffee [2]. By contrast, analyses of NHANES data for years 2011–2016 showed than only 20.8% of adults aged >20 years drank tea [3]. Mean coffee consumption among coffee drinkers was estimated at 417 mL/day [2]; mean tea consumption among tea drinkers was estimated at 91.6 g/day [3,4]. Water consumption easily exceeded that of coffee and tea combined. In the NHANES 2015–2016 data, mean intake of plain drinking water, bottled and tap, among adults was estimated at 1271 mL/day [3]. 

The consumption of plain water, coffee, and tea in the US varied with age, race/ethnicity, education, and incomes [2,3,4]. In the NHANES 2003–2012 data, coffee consumption was higher in older age groups and among non-Hispanic Whites [2]. No effects of education or income were observed [2]. In the NHANES 2011–2016 data, tea consumption was higher in older age groups and among non-Hispanic Whites and Asians than among non-Hispanic Blacks or other groups [4]. Both tea consumption and flavonoid intakes were linked to higher socio-economic status [4,5]. Interestingly, a socioeconomic gradient was also observed for the consumption of plain water from the tap [3]. In the NHANES 2011–2016 data, tap water intake was higher among non-Hispanic Whites and higher income groups [3]. 

The current socioeconomic characteristics of coffee consumption in the US have been difficult to pin down. Some studies of beverage consumption trends in the US have aggregated brewed tea and coffee into a single category [6]. Exposure was defined in terms of high coffee consumption versus low (or no) coffee consumption [7]. Elsewhere, coffee consumers in NHANES 2003–2012 data were identified using a combination of food frequency questionnaires and 24-h dietary recalls [2]. Socio-economic status was dichotomized into more than high school diploma or high school diploma or less [2]. Numerous other studies have focused on caffeine intakes as opposed to the consumption of coffee and coffee beverages [8,9,10]. Although coffee is the largest single source of caffeine by far, caffeine is also present in sweetened beverages and in energy drinks [11]. 

Consumption surveys conducted by the coffee industry suggest that 64% of Americans aged 18 years and older drink a cup of coffee every day, and 79% of them drink coffee at home [12]. Coffee consumption increased with age, with 72% of adults over 60 y classed as coffee drinkers [12]. Workplace consumption has increased from 16% in 2013 to 21% in 2015 according to the National Coffee Data Trends Report that is available for purchase [13]. Consumer panel data also suggest that the younger demographics consume more coffee away from home [12,13].

The present analyses examined whether similar trends would be found in multiple years of the NHANES data. As the premier US program for monitoring population diet [14], the NHANES serves as the evidence base for nation-wide dietary recommendations and guidelines [15]. This study used dietary data from NHANES 2011–2016 to explore coffee consumption patterns across different socio-demographic groups and by purchase or sourcing location [16,17,18]. 

Given that foods and beverages are consumed as part of dietary patterns, the Dietary Guidelines 2015–2020 [15] raised the issue of whether coffee consumption was associated with additional calories from milk, cream, added sugars, or other additions that would need to be accounted for within the eating pattern. Secondary analyses addressed coffee consumption patterns in relation to sugar and fat intakes, compliance with the Dietary Guidelines for Americans, and other diet quality measures [4,5,6]. 

## 2. Materials and Methods 

### 2.1. Dietary Intake Databases

Consumption data for coffee and coffee beverages came from 3 cycles of the National Health and Nutrition Examination Survey (NHANES), for the years 2011–2012, 2013–2014, and 2015–2016 [14]. The three NHANES cycles provided a total sample of 29,902 persons. The present analyses were based on 14,865 persons who were aged ≥20 years and had at least one valid 24 h dietary recall as defined by NHCS staff. Two-day means for all dietary variables were calculated for respondents with two 24 h dietary recalls, in line with previously published methods [19]. The present analyses were based on 8551 persons who were aged ≥20 years and had consumed any amount of coffee on one or both of their recall days. 

The 24 h recall in NHANES studies is administered by trained interviewers, using a computerized interface. Participants are asked to report the types and amounts of all food and beverages consumed in the preceding 24 h, from midnight to midnight [20]. The multi-pass method had respondents first identify a quick list of foods and beverages consumed. The time and consumption occasion for each food item were also obtained. The following passes asked for amounts consumed, and a final probe asked for any forgotten foods. Day 1 interviews were conducted in a mobile examination setting. Day 2 interviews were done by telephone some days later [21]. The mean number of days between the two dietary recalls was 7.9 days (median 5 days).

### 2.2. Participant Characteristics

NHANES participants were stratified by age, gender, education, family income-to-poverty ratio (IPR) and race/ethnicity. The age group cut points were: 20–30, 31–50, 51–70, and >70 years. Gender was defined as male/female. Education was defined as <high school; high school; some college; and completed college. Family IPR is the ratio of family income to the federal poverty threshold. In 2016, federal poverty level was defined as annual income of USD 24,300 for a family of 4. The cut points for IPR were <1; 1–1.99; 2–3.49; and ≥3.5. Race/ethnicity was defined as non-Hispanic White; non-Hispanic Black, Mexican American, Other Hispanic, non-Hispanic Asian, and other/mixed race. 

### 2.3. Defining Coffee Consumers

The NHANES 24-h recall data provide the amounts in grams of each food and beverage consumed [14]. Coffee consumption was defined based on the What We Eat in America (WWEIA) Food Categorization scheme, which breaks all foods consumed into 155 categories. A few non-coffee containing beverages included in the WWEIA coffee category were excluded (e.g., postum and chicory). Coffee consumers were identified as those NHANES participants who had consumed any amount of coffee on one or both of their recall days. Based on these criteria, the final sample of coffee consumers was 8551. 

Given that the place where the coffee was prepared or purchased was of interest, we excluded the small number of ready-to-drink canned/bottled coffees (<0.5% of all consumed coffee). 

### 2.4. Defining Source Locations for Coffees

For each food/beverage in the 24-h recall, NHANES respondents also reported the source location. Those were assigned in the database into 26 categories including store, fast food restaurant, full-service restaurant, convenience store, common coffee pot, from someone else/gift, and mail order, among others. 

The present analyses were based on 8 distinct sources. Those were: stores (including mail order), fast-food restaurants, convenience stores, from someone else/gift, restaurant, common coffee pot, cafeteria, and an additional other category. Coffee from someone else normally referred to drinking coffee at somebody’s house, away from home. In rare instances where the coffee and coffee additions came from difference sources, the source of the coffee was the default option. The place of consumption (home vs. away from home) was used as an additional variable. As individuals could obtain coffee from multiple sources, we estimated the mean grams of coffee (and additions) from each source and at the population-level estimated the population ratio of coffee consumption from that source.

### 2.5. Diet Quality Indicators

The energy and nutrient content of the diet were calculated using the USDA Food and Nutrient Database for Dietary Studies (FNDDS) [22]. Data from the Food Patterns Equivalents Database (FPED) from the United States Department of Agriculture (USDA) [23] were used to estimate intakes of food groups (e.g., vegetables or fruit) in order to calculate Healthy Eating Index HEI-2015 scores. 

The HEI-2015 [24] is an energy-adjusted measure of diet quality that is based on the intake of 9 food groups/nutrients to encourage and 4 food groups/nutrients to limit. Total fruits, whole fruits, total vegetables, greens and beans, whole grains, dairy, total protein foods, seafood and plant protein, and fatty acids ratio are the 9 items to encourage. Refined grains, sodium, added sugars and saturated fat are the 3 items to limit. The HEI-2015 is an operationalized measure of compliance with the 2015–2020 Dietary Guidelines for Americans.

The second measure of diet quality was provided by the Nutrient-Rich Foods (NRF9.3) index [25]. The NRF9.3 score is based on two subscores: the positive nutrient rich (NR) subscore and the negative nutrients to limit (LIM) subscore. The NR subscore contains 9 nutrients to encourage whereas the LIM subscore contains 3 nutrients to limit. The US Food and Drug Administration (FDA) and other standards were used to establish daily values, expressed as percentages. The reference amounts were: protein (50g), fiber (28 g), calcium (1300 mg), iron (18 mg), potassium (4700 mg), magnesium (420 mg), vitamin A (900 RAE), vitamin C (90 mg), and vitamin D (20 mcg). For the 3 nutrients to limit the maximum recommended values (MRVs) were: saturated fat (20 g), added sugar (50 g) and sodium (2300 mg). The NRF9.3 formula was calculated as:

NRF9.3 = (NR9 − LIM) × 100

where

(1) NR=∑i=19IntakeiEnergy×2000DVi

(2) LIM=∑i=13IntakeiEnergy×2000MRVi−1

where *Intake_i_* is the daily intake of each nutrient *i* and *DV_i_* is the reference daily value for that nutrient. In calculating the NR subscore, each daily nutrient intake *i* was adjusted for 2000 kcal and expressed in percentage of DV. Following past protocol, percent DVs for nutrients were truncated at 100, so that an excessively high intake of one nutrient could not compensate for the dietary inadequacy of another. In calculating LIM, only the share in excess of the recommended amount was considered. 

### 2.6. Data Availability and Ethical Approval

The National Center for Health Statistics (NCHS) and its Institutional Review Board (IRB) provided the needed approval for NHANES protocols [26]. Written informed consent was provided by adult participants. All NHANES data are publicly available and posed on the NCHS and USDA websites [27]. According to the University of Washington (UW) policies, analyses of public data do not involve “human subjects” and do not require IRB review or an exempt determination. Such data may be used and analyzed without any involvement of the Human Subjects Division or the UW Institutional Review Board.

### 2.7. Statistical Analyses

The survey-weighted mean intakes of coffee were evaluated overall and by gender, age group, race/ethnicity, family IPR and education. Energy and nutrient intakes of coffee consumers (n = 8551) were compared to those of non-consumers (n = 6314). Similar analyses were used to examine coffee consumption broken into survey-weighted tertiles with a fourth category for non-coffee consumers. 

Differences between survey-weighted means and proportions were tested using Wald tests and multivariable adjusted analyses were conducted using survey-weighted linear regression models with adjusted means represented as the marginal mean holding all covariates fixed as the approximate population-level average. Levels of statistical significance are indicated in the tables. All analyses accounted for the complex survey design of NHANES and reflect dietary behaviors of the US adult population from 2011–2016. All analyses were conducted using Stata 16.0 (College Station, TX, USA).

## 3. Results

### 3.1. Characteristics of Coffee Consumption among Adults Aged > 20 years 

Coffee consumers were those NHANES 2011–2016 participants who drank coffee only on day one (6.1% of those with two recalls), only on day two (6.6% of sample with two recalls), or on both days (48.3% of the sample with two recalls). The percent of coffee consumers aged >20 y in the total sample was 59.5% and the mean amount consumed was 324 g/day or 10.9 fluid oz. 

Table 1 shows percent consumers and amounts consumed by socio-demographic characteristics of the NHANES sample. Age group and race/ethnicity had significant effects on percent consumers and amounts consumed. Among those 20–30 years, 38.9% drank coffee and the mean amount consumed was 164 g/day (5.5 fluid ounces). In the ≥71 years age group, 74.3% drank coffee and the mean amount consumed was 361 g/day (12.2 fluid ounces). Among non-Hispanic Whites, 63.7% drank coffee and the mean amount consumed was 386 g/day (13.0 fluid ounces). Among non-Hispanic Blacks, 37.7% drank coffee and the mean amount consumed was 140 g/d (4.7 fluid ounces). Coffee consumption was higher at higher incomes. For lower income individuals below federal poverty threshold (IPR < 1.00), prevalence of consumption was 50.3% and the mean amount was 252 g/day. (8.5 fluid ounces). For higher income individuals (IPR > 3.5), the prevalence of consumption was 64.4% and the mean amount consumed was 316 g/day. The prevalence of consumption was the same for women and men but men drank more coffee. No effect of education on the amount of coffee consumed was observed.

### 3.2. Coffee Consumers versus Non Consumers

Among adults identified as coffee consumers (n = 8551), the average amount of coffee consumed was 544.7 g/day or 18.3 oz. Median consumption was 439 g/day (14.8 oz) and interquartile intervals were 265–690 g/day (8.9 oz to 23.2 oz). Amounts of coffee consumed varied across population subgroups. Men consumed more coffee on average than did women. The effect of age was significant (*p* < 0.001); coffee consumption peaked for the 51–70 y age group (620.4 g/day) and declined for the ≥71 years age group (486 g/day). The amounts of coffee consumed also varied by race/ethnicity (*p* < 0.001). The non-Hispanic white population consumed most coffee (605.5 g/day or 20.3 oz). The least coffee was consumed by non-Hispanic Black (371.8 g/day) and non-Hispanic Asian groups (368.5 g/day or 12.4 oz). 

Table 2 shows energy and nutrient intakes together with overall dietary quality scores for consumers and non-consumers of coffee. Nutrient values were adjusted for energy (Model 1) and multivariable analyses adjusted energy, age group, gender, race/ethnicity and family income to poverty ratio (Model 2). Energy and protein intakes of coffee consumers and non-consumers were not significantly different adjusting for covariates. Coffee consumers had diets with significantly less carbohydrate and less added sugar. However, their diets were higher in total fat (*p* < 0.01), PUFA (*p* < 0.05), MUFA (*p* < 0.01), saturated fat (*p* < 0.05), solid fat (*p* < 0.01) and cholesterol (*p* < 0.001). The observed differences in dietary fat content of the diet between coffee consumers and non-consumers, though statistically significant, were negligible (Total fat: 1.2 g, PUFA: 0.3 g, MUFA: 0.3 g, SFA: 0.4 g).

Coffee consumers had diets with more potassium and magnesium (*p* < 0.001) but less vitamin C (*p* < 0.001). No differences for calcium and vitamin D were observed. Mean caffeine consumption was 233 mg/day for consumers and 72.3 mg/day for non-consumers (*p* < 0.001). Coffee consumers had higher Nutrient-Rich Foods (NRF9.3) scores than did non consumers: however, this difference was attenuated in adjusted models. Coffee consumers had higher Healthy Eating Index (HEI-2015) scores in the energy-adjusted analyses and the effect remained significant in the multivariable model (*p* < 0.01).

Table 3 shows energy, nutrient intakes, and diet quality scores by tertiles of coffee consumption with non-consumers as an additional category. Nutrient values were adjusted for energy, age, gender, race/ethnicity and family income to poverty ratio. Protein intakes were not significantly different by trend analyses. Total carbohydrates (trend *p* < 0.001) and added sugars (trend *p* < 0.01) declined significantly with increasing coffee consumption. Higher coffee consumption was associated with diets that were higher in total fat (*p* < 0.001), PUFA (*p* < 0.001), MUFA (*p* < 0.001), solid fat (*p* < 0.001) and cholesterol (*p* < 0.01). Higher coffee consumption was associated with diets that were higher in potassium and magnesium (*p* < 0.001) but lower in vitamin C (*p* < 0.001). No differences for calcium and vitamin D were observed. Mean caffeine consumption increased from 125 to 380 mg/day on going from the first to the third tertile of coffee consumption.

In contrast to the comparison of consumers and non-consumers, the amount of coffee consumed had little to no impact on Nutrient-Rich Foods (NRF9.3) or on Healthy Eating Index (HEI-2015) scores.

### 3.3. Coffee Source Locations by Age and Socio-Demographics

**Figure 1:**
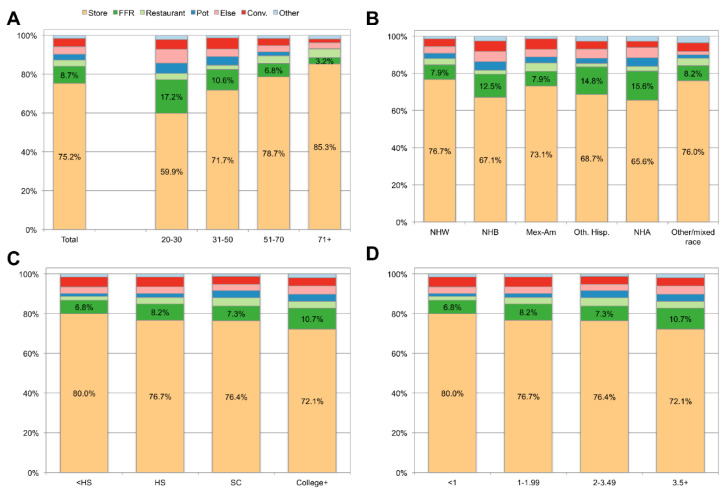
Sources of coffee overall and by age group (Panel **A**), race/ethnicity (Panel **B**), education (Panel **C**), and family income-to-poverty ratio (Panel **D**). Acronyms: FFR stands for fast food restaurant, Pot stands for common coffee pot, Conv stands for convenience store; NHW stands for non-Hispanic White, NHB stands for non-Hispanic Black, Mex-Am stands for Mexican-American, Oth. Hisp. stands for other Hispanic, NHA stands for non-Hispanic Asian, HS stands for HS, SC stands for Some college.

Figure 1 shows the distribution of coffee source locations in the total population overall and by age group, race/ethnicity, education and family income. In the total population, 75.2% of coffee came from stores, 8.7% from fast food restaurants, which includes quick-service coffee shops, 4.3% from convenience stores, 3.8% from someone else (e.g., gift), 3.4% from restaurants, 2.9% from a common coffee pot, and 1.6% from other sources. 

Across all groups, grocery stores were the most common source of coffee (>59% in all groups), but distinct purchase patterns by location and population subgroup were also observed. Among older adults, more than 85% of coffee came from grocery stores as compared to 59% among younger adults. Younger adults were more likely to obtain coffee from fast food restaurants, convenience stores, someone else, cafeteria, and common coffee pot. Among younger adults, fast food restaurants contributed 17.2% of coffee, as compared to only 3.2% amongst the oldest adults. 

Some differences were also observed by race/ethnicity. Most likely to get coffee from stores were non-Hispanic Whites (75.7%), Mexican Americans (73.6), Hispanic (72.3%) and other groups (77%). Least likely were non-Hispanic Black (63.0) and non-Hispanic Asian (63.8%) groups. Non-Hispanic Black adults were more likely to get coffee from fast food restaurants and convenience stores as compared to non-Hispanic White adults. Groups with more education and with higher incomes tended to get coffee from fast food restaurants more than other groups and those with less education were more likely to get coffee from convenience stores. Data by gender are not shown in the graphs, but women were more likely to get coffee from stores (77.7% for women compared to 73.1% for men). Men were more likely to take coffee from a common coffee pot than women (3.9% for men vs. 1.8% for women).

## 4. Discussion

Based on the 2015–2020 Dietary Guidelines [15], moderate coffee consumption—up to three to five 8-oz cups/day containing up to 400 mg/day of caffeine—can be incorporated into healthy eating styles. Those levels of coffee consumption (740 to 1200 mL/day) have not been associated with an increased risk of premature death, especially from cardiovascular disease, or with higher mortality from cancer [15]. The present analyses showed that mean coffee consumption among coffee drinkers was 544.7 g/day (18.3 oz), well within the DGA guidelines for a healthy diet. 

About 59% of NHANES 2011–2016 participants consumed coffee on one or both days of dietary data collection. These data contrast with an estimated of 79% based on 2003–2006 NHANES. However, Loftfield et al. [3] used a broader definition, identifying coffee drinkers as those who reported ever consuming coffee in the previous 12 months on the FFQ or reported coffee intake on one or more 24-h dietary recalls. Using different income cut points, the present findings confirmed a significant effect of income that applied not only to coffee consumption but also to coffee purchase location. Most of the coffee (74%) was purchased in grocery stores for preparation and consumption at home. 

The present analyses of nationally representative NHANES data are consistent with 2018 industry reports that 64% of Americans aged ≥18 years said they had a cup of coffee the previous day [12,13]. The figure for 2017 was 62%, according to results of a survey commissioned by the National Coffee Association (NCA) and available for purchase. The present finding that away-from-home coffee was more typical of younger participants and groups with higher education and incomes was also consistent with industry reports [12,13].

The current consensus is the “healthy” beverages are plain drinking water and coffee and tea without added sweeteners. Each of those beverages follows distinct patterns of consumption [4,5]. The present data provide a direct comparison with previously published analyses of both water and tea consumption patterns among US adults in the same NHANES 2011–2016 data [4]. Compared to widespread water and coffee consumption, only 20.8% of adults were tea consumers [4]. For both coffee and tea, percent consumers and amounts consumed increased with age. However, whereas 74% of adults in the >70 age group drank coffee, only 27% in that age group drank tea. For coffee, the effect of income was significant but the effect of education was not. For tea, the effects of both income and education were significant [4]. For both tea and coffee there were significant effects of race/ethnicity. Coffee and tea consumption were higher among non-Hispanic Whites than among non-Hispanic Blacks and other groups.

A social gradient was observed for drinking water. Higher tap water consumption was associated with higher incomes, but bottled water was not. Non-Hispanic Whites consumed most tap water (781 mL/day), and Mexican Americans consumed most bottled water (605 mL/day). Whereas the US consumption patterns for drinking water, coffee, tea and diet soft drinks appear to be associated with higher socioeconomic status, the consumption of caloric sugar sweetened beverages follows the opposite social gradient [16].

Identifying the coffee purchase location was a novel component of this study [16,17,18]. About 74% of coffee was purchased in stores to be prepared and consumed at home. This finding is also consistent with industry sources showing that at-home preparation continues to be dominant [12,13]. We now provide additional data showing that consumption patterns for coffee consumed at cafes and other out-of-home locations varied by age, income, and race/ethnicity.

The present estimates of dietary nutrient density among coffee consumers and non-consumers provide some parallels as well as contrasts with tea consumption. Coffee consumption among adults in the 2011–2016 NHANES sample was associated with greater compliance with the 2015–2020 Dietary Guidelines for Americans, as assessed by higher HEI-2015 scores [25]. Each 3% reduction in total dietary energy from empty calories leads to a 2-point improvement in HEI-2015. However, the observed effects of coffee on diet quality were not nearly as pronounced as those that had previously been observed for tea. Tea consumers in the same 2011–2016 NHANES database had diets that were lower in added sugars but higher in protein, vitamins and minerals. Coffee consumers had diets that were lower in total carbohydrates and in added sugars but were higher in total fats. Diets of coffee consumers were higher and potassium and magnesium (coffee is a top dietary source of potassium after milk) but they were not higher in calcium or vitamin D as compared to the non-consumers Consistent with past reports, diets of coffee consumers were higher in alcohol. The overall effect on HEI 2015 scores was weak, although it remained significant after adjusting for covariates.

The Dietary Guidelines 2015–2020 noted that while coffee consumption was not detrimental to health, coffee beverages may include calories from added sugars and/or saturated fat (such as cream, whole or 2% milk, and creamer), both of which should be limited [15]. The present analyses showing that the diets of coffee consumers were actually lower in added sugar ought to ease the concerns expressed in Dietary Guidelines 2015–2020. It may be that coffee drinkers consumed less sugar sweetened beverages (SSB) due to their more advanced age and higher socioeconomic status. For those groups, caloric SSB may have been replaced by coffee. However, diets of coffee consumers were also higher in total fats including MUFA and PUFA but also saturated and solid fats and cholesterol. Calcium intakes of which milk is a main source did not differ between coffee consumers and non-consumers. The present association between caffeine intakes and alcohol consumption has been observed before in published analyses of the NHANES 2007–2012 data [9].

The present study had limitations. The NHANES studies are based on a cross-sectional design, which means that causality cannot be inferred. NHANES 24 h dietary recalls were based on self-report. Assessments of dietary nutrient density lacked novel data bases such as polyphenols. Whereas tea is the main source of bioactive flavonoids [5], coffee along with wine is among the main sources of dietary polyphenols [28,29]. Nevertheless, the scale and representativeness of the NHANES sample make it the premier study of dietary intakes in the US and the foundation of food and nutrition policy.

## 5. Conclusions

The present analyses provide additional evidence regarding the relation between the consumption of beverages and achieving nutrient and food-based dietary guidelines. Mean coffee consumption among coffee consumers was within the DGA guidelines as were the mean intakes of caffeine. Among adults, the consumption of coffee, tea, and drinking water was associated with improved dietary nutrient density and higher incomes. A polyphenol database may prove useful for designing a new guidance system for beverage consumption in the US [30].

## Figures and Tables

**Table 1:** Sample characteristics for all adults (age ≥ 20 years) in NHANES, 2011–2016.

	n	% Consumers	Mean g/day
Total	14,865	59.5	324
Age group			
20–30	2850	38.9	164
31–50	5071	57.8	308
51–70	4873	69.2	430
≥71	2071	74.3	361
*p*-value		<0.001	<0.001
Gender			
Male	7223	58.7	357
Female	7642	60.3	293
*p*-value		0.18	<0.001
Race/ethnicity			
Non-Hispanic white	5786	63.7	386
Non-Hispanic black	3343	37.7	140
Mexican-American	2010	58.3	238
Other Hispanic	1590	64.5	248
Non-Hispanic Asian	1656	50.8	187
Other/mixed race	480	59.9	325
*p*-value		<0.001	<0.001
Education ^a^			
<High school	3280	60.0	307
High school	3257	57.7	319
Some college	4533	56.8	323
≥College	3787	63.4	338
*p*-value		0.002	0.39
Family income-to-poverty ^b^			
<1.00	3119	50.3	252
1.00–1.99	3601	56.0	287
2.00–3.49	2836	60.3	340
≥3.5	4101	64.4	364
Missing	1208	59.7	316
*p*-value		<0.001	<0.001

^a^ There were eight individuals missing education data. ^b^ The IPR accounts for household size. Individuals with missing IPR data are excluded.

**Table 2:** Energy and nutrient intakes and nutrient density/diet quality scores (NRF and HEI) for coffee consumers (N = 8551) and non-consumers (N = 6314), NHANES 2011–2016.

	Mean (95% CI)			
	Model 1 Energy-Adjusted ^a^		Model 2 Multivariable-Adjusted ^b^	
	Coffee Consumer (n = 8551)	Non-Consumer (n = 6314)	Coffee Consumer (n = 8551)	Non-Consumer (n = 6314)
Calories, kcal/day	2065 (2039, 2091) ***	2118 (2091, 2145)	2089 (2068, 2111)	2082 (2061, 2105)
Macronutrients				
Protein, g/day	81.2 (80.2, 82.2)	79.9 (78.9, 81)	81.1 (80.2, 82)	80 (78.9, 81.1)
Carbohydrate, g/day	235.4 (233.4, 237.4) ***	244.4 (242.3, 246.6)	235.7 (234.1, 237.3) ***	244.1 (241.7, 246.4)
Added sugar, teaspoon/day	14.6 (14.2, 15) ***	16.5 (16, 17)	14.8 (14.5, 15.2) ***	16.1 (15.6, 16.6)
Total fat, g/day	77.2 (76.6, 77.9) ***	75.5 (74.7, 76.2)	77 (76.5, 77.6) **	75.8 (75, 76.6)
PUFA, g/day	24.9 (24.7, 25.2) ***	24.3 (24, 24.7)	24.8 (24.6, 25) *	24.5 (24.2, 24.8)
MUFA, g/day	27.2 (27, 27.5) ***	26.4 (26.1, 26.7)	27.2 (26.9, 27.4) **	26.5 (26.2, 26.9)
SFA, g/day	18.2 (18, 18.4) ***	17.7 (17.5, 18)	18.2 (18, 18.3) *	17.8 (17.6, 18.1)
Solid fat, g/day	32.8 (32.3, 33.3) ***	31.7 (31.1, 32.2)	32.7 (32.3, 33.2) **	31.8 (31.3, 32.3)
Other dietary constituents				
Caffeine, mg/day	238.8 (230.6, 247.0) ***	63.9 (57.9, 69.8) ***	233.0 (226.5, 239.6) ***	72.3 (66.4, 78.2)
Calcium, mg/day	948.3 (935.4, 961.2)	941.8 (920, 963.6)	940.5 (928.1, 953)	953.2 (933.1, 973.2)
Potassium, mg/day	2787.6 (2753.4, 2821.7) ***	2466.6 (2429.1, 2504.1)	2742.2 (2713.9, 2770.5) ***	2533.2 (2497.4, 2569.1)
Magnesium, mg/day	313.2 (308.3, 318.1) ***	285.7 (281.2, 290.3)	309.4 (305, 313.8) ***	291.3 (286.5, 296)
Vitamin C, mg/day	81.3 (78.5, 84.1) **	86.4 (82.4, 90.5)	80 (77.5, 82.4) ***	88.4 (84.3, 92.6)
Vitamin D, mcg/day	4.8 (4.6, 4.9)	4.6 (4.4, 4.8)	4.7 (4.5, 4.8)	4.7 (4.5, 4.9)
Sodium, mg/day	3397.3 (3365.5, 3429)	3436.6 (3397.5, 3475.7)	3400.7 (3372.1, 3429.3)	3431.6 (3393.4, 3469.7)
Cholesterol, mcg/day	290.8 (284.5, 297) ***	273.9 (268.8, 279.1)	291.1 (285.1, 297) ***	273.5 (267.9, 279.1)
Alcohol, g/day	9.5 (8.7, 10.3) ***	7.1 (6.4, 7.8)	9.6 (8.9, 10.2) ***	7 (6.2, 7.8)
Nutrient density/diet quality				
NRF9.3	436.2 (430.2, 442.1) ***	414.3 (407.8, 420.7)	430 (424.7, 435.2)	423.4 (416.9, 429.9)
HEI-2015	52.9 (52.3, 53.5) ***	50.4 (49.8, 50.9)	52.3 (51.9, 52.8) **	51.2 (50.6, 51.8)

Abbreviations: MUFA monounsaturated fatty acids; PUFA polyunsaturated fatty acids; SFA saturated fatty acids; NRF Nutrient Rich Food Index; HEI-2015 Healthy Eating Index. ^a^ Adjusted for energy. Analyses of energy are not energy-adjusted. ^b^ Adjusted for energy, age, gender, race/ethnicity and family income to poverty ratio. Asterisks indicate *p*-value comparing coffee consumers to non-consumers; *** *p* < 0.001; ** 0.001 < *p* < 0.01; * 0.01 < *p* < 0.05.

**Table 3:** Association between amount of coffee consumed (survey-weighted tertiles) and diet composition and diet quality measures.

	Mean (95% CI)^a^				*p*-Trend
	Non-Consumers (n = 6314)	T1 [1.3–319.2 g/day] (n = 3532)	T2 [319.3–585 g/day] (n = 2834)	T3 [≥585.2 g/day] (n = 2185)		
Calories, kcal/day	2080 (2057, 2103)	1997.6 (1967, 2029)	2103.9 (2063, 2145)	2172.7 (2134, 2211)	<0.001
Macronutrients					
Protein, g/day	80.1 (79, 81.2)	81.6 (80.2, 83)	81.2 (79.8, 82.6)	80.5 (79.3, 81.6)	0.484
Carbohydrate, g/day	244.2 (241.8, 246.5)	238.7 (236.4, 241.1)	235.6 (232.5, 238.7)	232.5 (229.9, 235)	<0.001
Added sugar, teaspoon/day	16.1 (15.6, 16.6)	14.6 (14, 15.2)	15 (14.5, 15.5)	15 (14.5, 15.4)	0.002
Total fat, g/day	75.8 (75, 76.6)	75.9 (75.1, 76.8)	77 (75.9, 78.1)	78.1 (77.2, 79.1)	<0.001
PUFA, g/day	24.5 (24.2, 24.8)	24.2 (23.8, 24.6)	24.9 (24.5, 25.2)	25.4 (25, 25.8)	<0.001
MUFA, g/day	26.5 (26.2, 26.9)	26.8 (26.4, 27.1)	27.1 (26.6, 27.6)	27.6 (27.2, 28.1)	<0.001
SFA, g/day	17.8 (17.5, 18.1)	18.1 (17.8, 18.4)	18.2 (17.8, 18.6)	18.2 (17.9, 18.5)	0.092
Solid fat, g/day	31.8 (31.3, 32.3)	31.3 (30.6, 32)	32.9 (32.3, 33.5)	34 (33.2, 34.9)	<0.001
Other dietary constituents					
Caffeine, mg/day	68.5 (62.7, 74.3)	125 (118.7, 131.4)	202.4 (196.4, 208.5)	380 (365.4, 394.7)	<0.001
Calcium, mg/day	953.2 (933.2, 973.3)	942.4 (920, 964.7)	939.7 (918.5, 961)	939.4 (915.3, 963.4)	0.333
Potassium, mg/day	2529.7 (2493.7, 2565.7)	2647.7 (2605.8, 2689.6)	2697.6 (2646.3, 2749)	2889 (2850.6, 2927.5)	<0.001
Magnesium, mg/day	291.1 (286.3, 295.8)	304.5 (297.9, 311.2)	307.1 (300.3, 313.8)	317 (311.6, 322.4)	<0.001
Vitamin C, mg/day	88.6 (84.4, 92.8)	84.8 (80.9, 88.7)	81.2 (76.5, 85.9)	73.6 (69.8, 77.4)	<0.001
Vitamin D, mcg/day	4.7 (4.5, 4.9)	4.8 (4.6, 5)	4.7 (4.4, 5)	4.5 (4.2, 4.7)	0.247
Sodium, mg/day	3432.8 (3394.6, 3470.9)	3445.2 (3396.1, 3494.2)	3371.5 (3325.1, 3417.9)	3383.1 (3325.9, 3440.3)	0.049
Cholesterol, mcg/day	273.5 (267.8, 279.1)	288.6 (279.2, 298)	292.9 (282.7, 303.2)	291.8 (281.8, 301.8)	0.002
Alcohol, g/day	7 (6.2, 7.8)	9 (8.2, 9.9)	9.6 (8.6, 10.6)	10.1 (8.9, 11.2)	<0.001
Nutrient density/diet quality					
NRF9.3	423.5 (417.1, 430)	435.6 (428.4, 442.8)	428.4 (420.4, 436.4)	425.5 (418.4, 432.6)	0.596
HEI-2015	51.2 (50.6, 51.8)	52.8 (52, 53.5)	52.4 (51.7, 53.1)	51.8 (51.1, 52.6)	0.076

Abbreviations: MUFA monounsaturated fatty acids; PUFA polyunsaturated fatty acids; SFA saturated fatty acids; NRF Nutrient Rich Food Index; HEI-2015 Healthy Eating Index. ^a^ Adjusted for energy, age, gender, race/ethnicity and family income to poverty ratio.
